# Using slow frame rate imaging to extract fast receptive fields

**DOI:** 10.1038/s41467-019-12974-0

**Published:** 2019-10-31

**Authors:** Omer Mano, Matthew S. Creamer, Catherine A. Matulis, Emilio Salazar-Gatzimas, Juyue Chen, Jacob A. Zavatone-Veth, Damon A. Clark

**Affiliations:** 1 Department of Molecular, Cellular, and Developmental Biology, Yale University, New Haven, CT 06511 USA; 2 Interdepartmental Neuroscience Program, Yale University, New Haven, CT 06511 USA; 3 Department of Physics, Yale University, New Haven, CT 06511 USA; 4 Department of Neuroscience, Yale University, New Haven, CT 06511 USA

**Keywords:** Sensory processing, Visual system

## Abstract

In functional imaging, large numbers of neurons are measured during sensory stimulation or behavior. This data can be used to map receptive fields that describe neural associations with stimuli or with behavior. The temporal resolution of these receptive fields has traditionally been limited by image acquisition rates. However, even when acquisitions scan slowly across a population of neurons, individual neurons may be measured at precisely known times. Here, we apply a method that leverages the timing of neural measurements to find receptive fields with temporal resolutions higher than the image acquisition rate. We use this temporal super-resolution method to resolve fast voltage and glutamate responses in visual neurons in *Drosophila* and to extract calcium receptive fields from cortical neurons in mammals. We provide code to easily apply this method to existing datasets. This method requires no specialized hardware and can be used with any optical indicator of neural activity.

## Abstract (web summary)

The temporal resolution of optical measurements of neural activity has traditionally been limited by the image or volume acquisition rate. Here, the authors describe an analysis that exploits the short duration of neural measurements within each image to extract neural responses at higher temporal resolution than the acquisition rate.

## Introduction

In investigating the function of circuits, experimenters often want to measure precise relationships between neural activity and other experimental variables, such as stimuli, behavior, or the activity of other neurons. One of the most effective ways to do this is with optical measurements of neural activity, since these record the dynamics of many neurons at once. These imaging techniques include scanning two-photon, light sheet, and laser scanning confocal microscopy. In these techniques, images are constructed from voxels (three-dimensional pixels) that are acquired sequentially. Because the individual voxels are acquired over short time intervals, each neuron is measured over a short period of time. Yet, because there are so many voxels to acquire, the period between measurements of a particular neuron can be much longer, equal to the duration of each full-image acquisition. Much effort has been invested in developing specialized hardware to increase the sampling rates of optical imaging^1–4^, yet imaging entire volumes (z stacks) frequently remains slow, with volumes typically acquired at ~10 Hz or slower^5–8^. Even when acquiring two-dimensional images, some imaging methodologies based on laser scanning (e.g. two-photon and confocal), may be too slow to capture fast dynamics of neural activity. Thus, imaging techniques often result in a set of neural measurements that are located precisely in time, but sampled infrequently, once per volume.

In contrast, other experimental variables can be sampled or presented much more frequently. Visual stimuli can be updated at 60 Hz or faster. Auditory stimuli may be modulated at frequencies of hundreds of Hz. Behaviors can often be measured from video recordings at 30 Hz or higher. And electrical signals like membrane potentials, local field potentials, or electromyograms are often recorded at 1 kHz or higher. These experimental variables are thus measured with high resolution in time.

The problem we address is how to relate neural activity that is sampled infrequently to other experimental variables that are sampled with high temporal resolution. In practice, when computing cross-correlations, temporal receptive fields, or peristimulus time histograms, such data has most often been analyzed by matching the effective sampling rates of the two variables. In some cases, experiments were explicitly designed to match the two sampling rates^9^. In other cases, rates have been matched after the experiment, either by downsampling the fast variable to match the infrequent neural measurements^5,8,10,11^ or by interpolating the infrequent measurements to match the high-resolution variable^12–17^. Both of these approaches suppress information at frequencies higher than the slower neural acquisition rate. The resulting cross-correlations and receptive field estimates are limited in their resolution by the Nyquist frequency, a well-established ceiling on the resolution of a measured response^18^. This is an unnecessary limit on the temporal resolution of these estimates.

Here, we apply methodology that exploits the precise timing of neural measurements within each infrequently-sampled frame to compute high temporal resolution relationships with frequently-sampled experimental variables, such as stimuli or behaviors. Since this method uses the timing of voxels acquired within each image, we refer to it as voxel-timing analysis. This method has been proposed for fMRI analysis^19,20^. Before that, similar methods were used to measure nuclear magnetic resonance responses with high temporal resolution^21^ and to achieve high temporal resolution in oscilloscopes^22^. It has been underappreciated how successfully voxel-timing analysis can be applied to cellular functional imaging studies. Using this analysis, the resolution of relationships between neural activity and other experimental variables is independent of the imaging frame rate. It is therefore not limited by the Nyquist frequency of the neural sampling interval. When signal properties are measured with resolution higher than the Nyquist frequency of the signal measurements, it is referred to as ‘super-resolution’^23–25^. Thus, voxel-timing analysis offers a way to achieve temporal super-resolution in relating neural activity to other experimental variables.

The principle behind this method can be illustrated with an analogy. Suppose we wish to record the trajectory of a ball that is bouncing after being dropped, but all we have is a camera that takes a photograph once every second. A single sequential set of images cannot capture the full trajectory of the ball, especially if the ball bounces faster than once per second. However, if we drop the ball repeatedly, then we can take a sequence of photographs with each drop. On each drop, we can introduce a different delay between the drop and the sequence of photographs. The result is several sequences of photographs, each representing the ball at a different set of points in its trajectory. Then, we may interleave photographs from many trials and reconstruct the complete trajectory of the bouncing ball. Thus, this method uses infrequent measurements (photographs once per second) to reconstruct a high-resolution trajectory of the bouncing ball. As we will show, this method can be formulated as a way to compute the precise cross-correlations (or receptive fields) between frequently and infrequently sampled variables. It is especially well-suited for typical neuroscience imaging data.

In this paper, we use simple synthetic examples to show how this method achieves temporal super-resolution with neural responses and to examine some trade-offs between the temporal resolution and noise in receptive fields measured using this method. We then apply this method to measure the response properties of neurons expressing three distinct indicators: voltage imaging in *Drosophila*, glutamate imaging in *Drosophila*, and calcium imaging in mammalian cortex. In all three cases, the voxel-timing method permits fast receptive fields to be computed, even when using slow acquisition rates. Interestingly, in some cases, infrequent measurements of neural activity can yield better filter estimates than an equivalent number of frequent measurements.

## Results

### Finding high-resolution filters from infrequent measurements

**Fig. 1:**
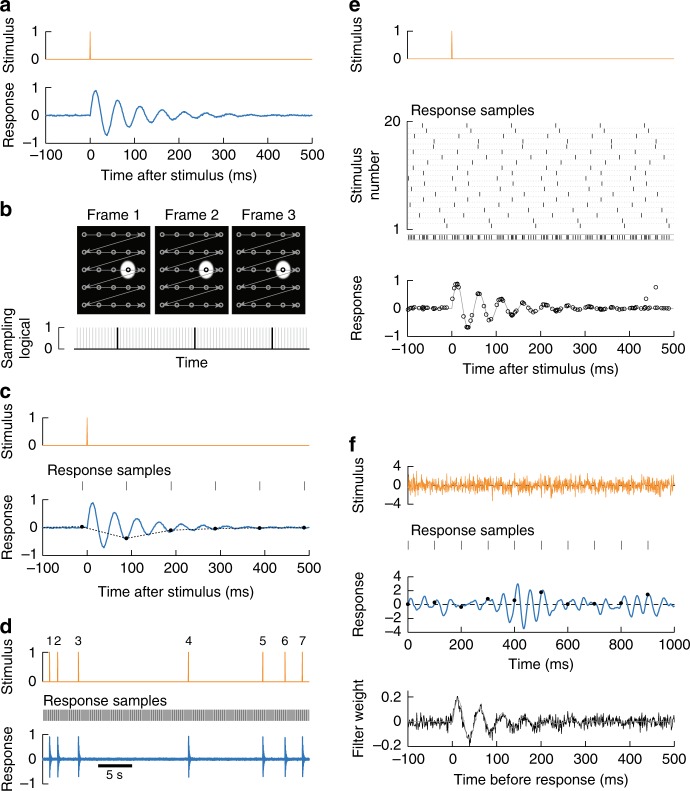
Precise responses from infrequent imaging measurements. **a** We simulated a neuron that can be stimulated with an impulse (top) and responds with damped oscillations at 20 Hz. **b** During the acquisition of many images, voxels are acquired serially, represented by the circles connected by arrows (top). Our simulated neuron, represented by the white circle, was only measured during a small fraction of the total frame acquisition. The black lines represent the sampling times of the neuron; gray lines represent all other samples in the image (bottom). The width of the black lines represents the voxel integration time, while the interval between them represents the frame duration. **c** Measurement of the response with infrequent sampling. When using an imaging scheme as in **b**, measurements of a single impulse (top) sample the response every 100 ms (middle). While the full response contains oscillations (bottom, blue), the sampled response shows no oscillations (circles), and interpolation cannot recover the true response (dashed line). **d** Sampled response measurements can be repeated across many stimulus impulses with random relative timing. **e** When many stimulus-response pairings are overlaid, the full, oscillating response may be recovered. Each individual stimulus is a very brief pulse (top). Each instance of the stimulus was aligned randomly with respect to the sampling intervals. Rasters show the sampling time of responses relative to each of 20 presentations of the stimulus, and the combination of all samples over all trials (middle). By overlaying each set of responses, without interpolating, the sampled responses (circles, bottom) reconstruct the true response (gray line). **f** The high-resolution impulse response may also be recovered when the stimulus is white noise, rather than impulses. Gaussian stimuli were presented to the same synthetic system as in **b** (top). The true response had a characteristic oscillation frequency (middle, blue), but infrequent sampling cannot resolve it finely (black circles). Using the precise timing of the samples, the cross-correlation between stimulus and response could be extracted with temporal super-resolution (bottom, black line), matching the true filter (gray line)

As a demonstration of how temporal super-resolution is achieved using voxel-timing, we considered a synthetic neural response to an impulse (Fig. 1a). In our simulation, impulse stimuli evoked a neural response that oscillated at 20 Hz before decaying back to baseline. A high-resolution measurement of the response would capture the full dynamics of its oscillation and decay.

However, imaging methods often do not provide continuous high-resolution measurements of the neural response. In neural-imaging methods where voxels are sampled serially, there are two timescales for each measurement. The first is the integration time of each individual voxel within a frame, while the second is the interval between successive measurements of the same voxel (the inverse of the frame rate) (Fig. 1b, Supplementary Fig. 1). Typically, the integration time is brief while the interval between measurements is long. We will leverage the short integration time to remove the resolution limitations normally imposed by the long intervals between measurements.

In our simulated experiment, we supposed that the response is sampled for 1 ms every 100 ms, due to sequential voxel acquisition. This corresponds to an integration time of 1 ms and a frame rate of 10 Hz. When the single trial response was sampled in this way, the oscillation in the response was not visible (Fig. 1c). Linearly interpolating the sampled response eliminated information about the oscillation (Fig. 1c, dashed line)^18^. However, even though the high-frequency response information was not available via interpolation, it did still exist within the sampled response^19^.

In the voxel-timing method, we took advantage of (a) the short voxel integration time; (b) the high resolution of the stimulus timing; and (c) the fact that the stimulus impulse may be presented many different times (Fig. 1d). The aim was to sample the response at every delay with respect to the stimulus by sampling responses with different offsets from the stimulus. The measured responses from all of the trials may be combined into a single trace to reconstruct the true, high-temporal resolution response (Fig. 1e).

As this example shows, obtaining high temporal resolution depends on sampling all relative delays between the high-resolution stimulus recording and the infrequent response measurements. Different delays could be intentionally engineered in an experiment, but when the imaging acquisition is not explicitly locked to the stimulus, the experiment will often sample all delays. To ensure this is the case, experimenters can choose a stimulus update rate and acquisition rate with no common integer multiples. Moreover, stimuli that are uncorrelated in time permit sampling of all possible delays regardless of stimuli and acquisition rates. Importantly, when using the voxel-timing method, the temporal resolution of the reconstructed response is independent of how frequently the response is sampled (see Supplementary Note 1). With enough data, any sampling rate can yield a good approximation to the true, high-resolution response.

As a first illustration, our example used widely-spaced impulse stimuli. In many experiments, stimuli are stochastic and continuously changing^26^, yet the same method can be applied. The precise timing of response measurements can be used to extract the high-resolution linear filter that best predicts the response from the stimulus (Fig. 1f). We discuss the details of this method in the next section.

### Procedure

The two examples shown in Fig. 1e, f are superficially quite different. In the first, one is computing the average response to precise events in the stimulus. In the second, one is computing the average stimulus weighted by the response at discrete points in time. In fact, both of these cases correspond to computing cross-correlations between a high temporal resolution variable (the stimulus) and one sampled infrequently but precisely in time (the response). Though there exist many sophisticated methods for inferring the structure of linear filters^27–31^, cross-correlations are simple and yield good intuition for the procedure.

**Fig. 2:**
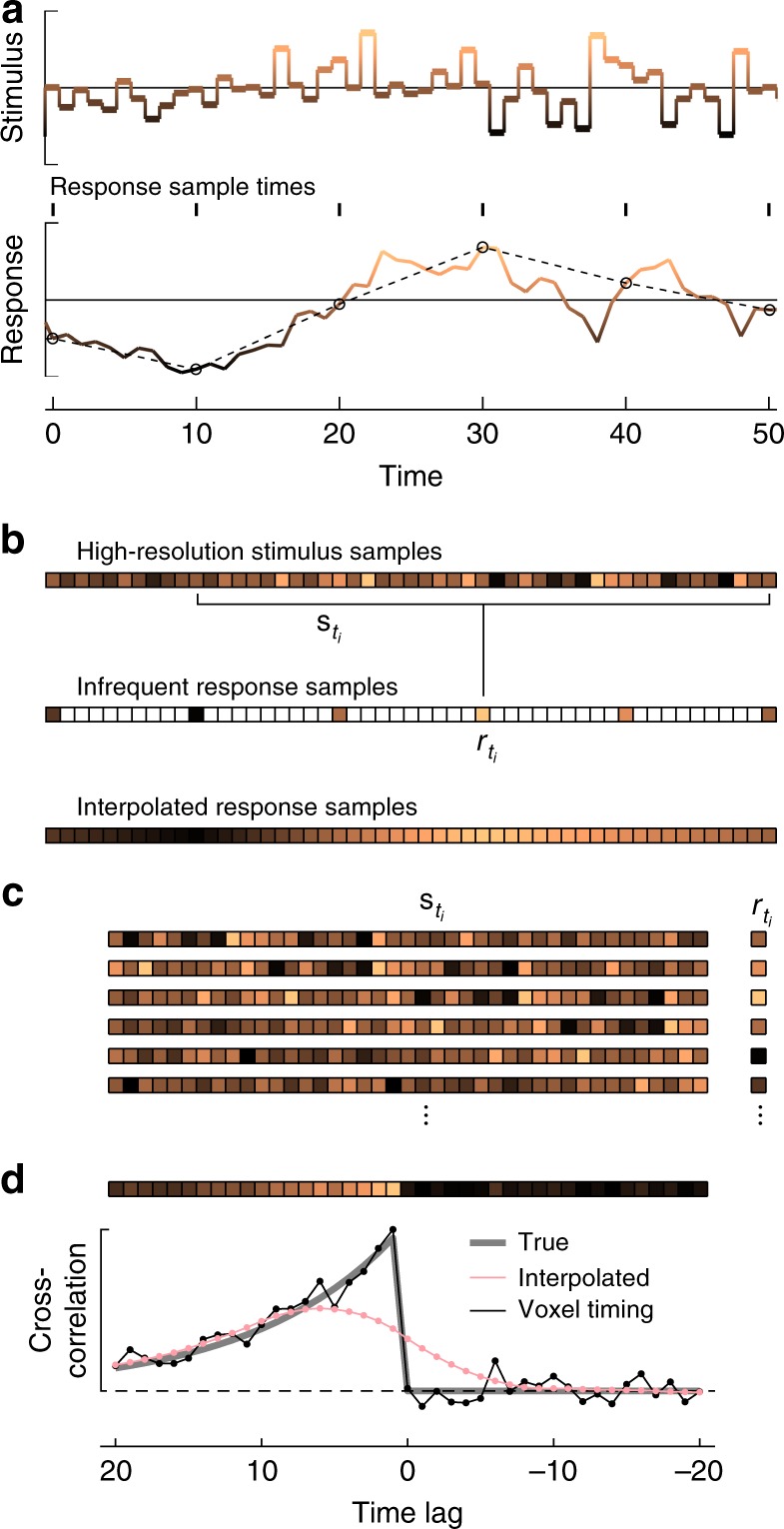
Procedure for computing temporal super-resolution cross-correlations. **a** Stimulus and response used in the numerical simulation. Stimulus values at each time were drawn from a Gaussian distribution. The response was computed as the convolution of the stimulus with an exponential filter with a timescale of 10 timesteps. Color and *y*-position each indicate the value of the functions. Responses were measured at the black circles; the dashed line represents a linearly interpolated response between measurements. **b** A vector representation of the stimulus may be constructed so that each element represents the value of the stimulus during that timestep (colors correspond to the value of the signals, as in **a**). A vector of the sampled response may also be constructed, leaving blank those elements where no measurements were made. The response was sampled every 10 timesteps. An alternate response vector may be constructed by interpolating the responses to generate response estimates during the frames when no measurements were made. For the sampled responses, if responses were measured at the set of times *t*_*i*_, then the response $r_{t_i}$ may be paired with a snippet of stimulus from the same region, $s_{t_i}$. **c** The set of (stimulus, response) pairings may be extracted. A simple analysis computes the cross-correlation between stimulus and response, which is an estimate of the linear filter that generates the response. **d** One finds the cross-correlation by multiplying the response with the stimulus and averaging over all measured responses. The cross-correlation estimate (represented both as a vector at top and a line plot at bottom) is a reasonable estimate of the true filter. When the response is interpolated, the high-resolution filter is not recovered. The time lag is defined as in the text and as in other figures

In this section, we show how to employ the voxel-timing method to compute a temporal super-resolution cross-correlation between the high-resolution stimulus and the infrequently sampled response (Fig. 2). As in Fig. 1, our simulated stimulus was recorded with high temporal resolution, and the response is sampled infrequently in time (Fig. 2a). First, one must construct a vector representation of the stimulus. Each element in this vector represents the stimulus at a single point in time at the rate of the stimulus update (Fig. 2b). One must then construct a vector representation of the response with the same high temporal resolution. Note that many elements in the response vector will be blank, since no measurements were made there. In the literature, response vectors have often instead been computed by interpolation, but we will show that interpolation generates a low-resolution filter estimate by implicitly smoothing the estimate. To understand the relationship between the high-resolution stimulus and the infrequently sampled response, we now pair vectors of the stimulus with specific responses (Fig. 2b, c). If the responses are measured at a set of times *t*_*i*_, then the pairings will be a set of stimulus vectors ${\boldsymbol{s}}_{t_i}$ and a set of responses $r_{t_i}$ (Fig. 2c).

To compute the cross-correlation between the variables, *c*, with a delay of *τ*, one multiplies each stimulus with that delay by the associated response, sums over all times *t*_*i*_, and then divides by the number of response measurements, *N* (Fig. 2d, see Supplementary Note 2):

$$c_\tau = \frac{1}{N}\mathop {\sum}\limits_{t_i} {s_{t_i - \tau }r_{t_i}}$$

Here, the correlation is a function of time lag, *τ*, which has increments equal to the spacing of the stimulus measurements. Both the stimulus and response are assumed to be mean subtracted. If one interpolates the response first, before computing the cross-correlation, the result is a smooth cross-correlation function with low resolution (Fig. 2d, see Supplementary Note 1). Instead, if one uses the voxel-timing approach, one recovers the true cross-correlation at high resolution (Fig. 2d).

If the stimulus is a set of impulses, such as in the example in Fig. 1d, then this equation is mathematically equivalent to a summation over those impulses, averaging the response at each delay. Thus, the computations in Fig. 1d, e are both cross-correlations, and they are equivalent.

The voxel-timing method can be used to measure relationships beyond the mean response to the stimulus, since it records entire distributions of responses aligned with stimuli. This can be important if one wishes to study the variability in the response. One way to analyze variability in the response is to extract second-order filters, which are the continuous-response equivalent of spike-triggered-covariance^32,33^. To demonstrate this technique, we modeled a cell that has a temporal receptive field that is modulated by a randomly changing amplitude (Supplementary Fig. 2a, b). This cell’s responses are widely variable because the sign of its receptive field changes throughout the simulation. In fact, the first-order filter is zero, independent of the sampling rate of the response (Supplementary Fig. 3c). Instead, one may compute the response-weighted stimulus covariance from the infrequently sampled data to show the correlations in the stimulus that result in large responses (Supplementary Fig. 2d). The first eigenvector of this matrix matches the underlying receptive field of the cell with resolution higher than the sampling rate (Supplementary Fig. 2e). Throughout this manuscript, we report estimated mean receptive fields, but voxel-timing analysis can be used in a variety of analyses to extract cellular properties with temporal resolution higher than the sampling rate.

### Noise, smoothing, and regularization

**Fig. 3:**
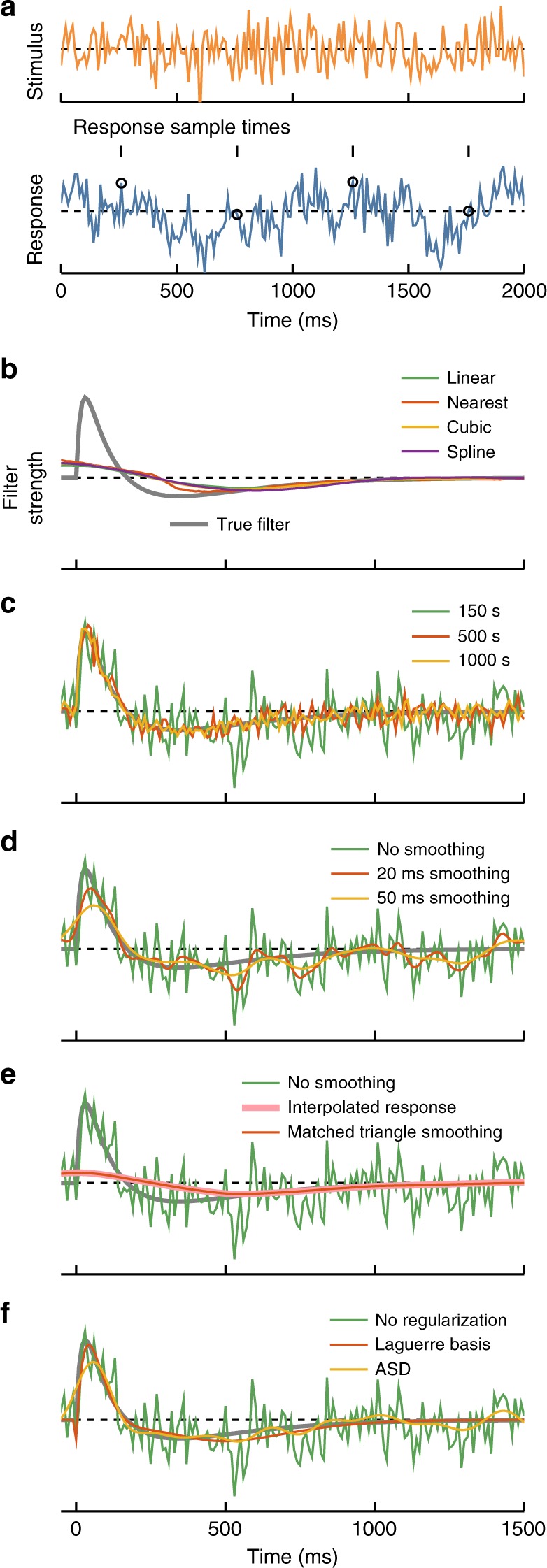
Noise in filter estimates. **a** Stimulus and response for these numerical experiments. The stimulus was updated from a Gaussian distribution every 10 ms. Responses were equal to a bilobed filter convolved with the stimulus. Responses included additive white noise, with a signal to noise ratio of 1. The response was sampled for 10 ms every 500 ms. **b** The true filter (gray) is compared to OLS filters extracted after upsampling the response using different interpolation methods: Linear interpolation, nearest neighbor, piecewise cubic, and spline interpolation. **c** Noise in the extracted best-fit filter decreased with increasing duration of the simulated experiment, due to an increasing number of samples. The filters are computed using the infrequently sampled response and OLS regression. **d** Noise in the extracted best-fit filter may also be reduced by smoothing in time. This trades off noise in the filter estimate for temporal resolution in the filter estimate. Here, the smoothing filters were Gaussian with the standard deviations noted. **e** By smoothing with an appropriate triangle filter, the temporal super-resolution filter may be transformed into the filter extracted from interpolated responses, i.e., the imaging-rate resolution filter. In this case, since the response samples are infrequent, the loss of resolution in the filter estimate is substantial. **f** Regularization methods may be applied to improve signal-to-noise in filter estimates. Here, the filter is fit in the five lowest-order terms of the discrete Laguerre polynomial basis or by using automatic smoothness determination (ASD)

In this section, we review the noise characteristics of the voxel-timing filter estimate that is found by ordinary least-squares (OLS) fitting of a linear weighting vector to predict each response, $r_{t_i}$, from each stimulus vector, ${\boldsymbol{s}}_{t_i}$. This is equal to the cross-correlation computed in Fig. 2 divided by the autocorrelation of the stimulus^27^ (see Supplementary Notes 1 and 2). When there are relatively few measured responses, this fitting procedure computes more accurate filters than simple cross-correlation, even for stimuli that are uncorrelated in time^34^. In this simulation, the neuron was measured for 10 ms every 500 ms. The stimulus was updated every 10 ms, so that the voxel-timing filter could have a resolution of 10 ms (Fig. 3a). Interpolating the response to match the stimulus sampling rate produces filter estimates that do not capture the dynamics of the true filter (Fig. 3b).

The temporal super-resolution voxel-timing filter has many parameters to estimate, one at each delay in the high-resolution vector. Each response measurement contributes a paired stimulus vector and response measurement to the cross-correlation computation. Thus, one expects the noise in the cross-correlation estimate to decrease like *N*^−1/2^, where *N* is the number of response samples (see Supplementary Note 3). In simulation, increasing the number of measured responses led to decreased errors in the filter estimate, as expected (Fig. 3c, Supplementary Fig. 3).

How else might one reduce the noise in the filter estimate? One simple method is to smooth the filter estimate in time. This averages over neighboring time points to decrease the noise in the estimate, but this decrease comes at the expense of temporal resolution (Fig. 3d)^35^. When one smooths over longer timescales, sharp features of the true filter are lost.

One can tune this temporal smoothing in order to trade off temporal resolution against noise. In particular, smoothing the voxel-timing filter with a triangle filter in time is equivalent to computing a filter from a linearly interpolated response signal (see Supplementary Note 1). The voxel-timing filter can be smoothed in a graded way to trade off noise for resolution (Fig. 3d) and can even be smoothed to precisely recover the low-resolution filter obtained by interpolating the response (Fig. 3e). This means that, given infrequent neural response measurements, there is little reason not to compute the voxel-timing filter.

A different way to reduce noise in filter estimates is to employ regularization techniques. These techniques constrain filter estimates by making assumptions about the form of the filter. They are easily applied to data sets of the form of pairs of stimuli and responses, ${\boldsymbol{s}}_{t_i}$ and $r_{t_i}$, and can be especially useful when data is limited. One technique is to fit the function in an alternative basis; a useful choice is the set of discrete Laguerre polynomials^36^. Fitting in this basis substantially reduced the noise in the filter estimate for the same number of response samples (Fig. 3f). This worked because the Laguerre basis provides an efficient representation of the true filter using only a few fit parameters. Another useful regularization technique is automatic smoothness determination (ASD), which ensures that the fitted filter is smooth^31,37^. ASD was also easily applied to this sort of data and reduced noise in the filter estimate (Fig. 3f). There are many other regularization techniques that can be applied in conjunction with this voxel-timing methodology^27–30^.

### Computer code

With this paper, we include code to generate all the simulation figures and the first application figure, so that readers may explore parameters most pertinent to specific experiments. We have also created a simple function that returns the voxel-timing cross-correlation, filter, or regularized filter. This makes it simple to apply this technique to existing and new datasets. The code is available at http://www.github.com/ClarkLabCode/FilterResolution.

### Applications to neural imaging data

To demonstrate the broad applicability of this method, we have employed it to measure properties of neurons in two different organisms expressing three common indicators of neural activity. First, we show how two-photon microscopy measurements can be used to extract fast receptive fields from *Drosophila* visual neurons expressing a voltage indicator. Second, we show how low frame rate two-photon imaging can be used to measure extracellular glutamate concentrations—a quantity only accessible by optical methods. Finally, we show how slow volumetric two-photon microscopy at 2 Hz or below could be used to extract temporal super-resolution receptive fields from GCaMP6s signals in tree shrew V1 neurons.

### High-resolution voltage filters from infrequent imaging

Precise timing of visual signals is critical to computing visual motion in vertebrates and invertebrates. In these circuits, motion is computed by delaying some visual signals relative to others^38,39^. Thus, measurements of filtering properties have been crucial to understanding motion computations in flies and in mammalian cortex and retina^12,40–50^. These filters may be measured precisely using optical voltage indicators, which report membrane voltage on fast timescales^13,51^. Here, we show that these voltage indicators can be used to extract high-resolution filters even when the frame rate of the imaging system is slow.

**Fig. 4:**
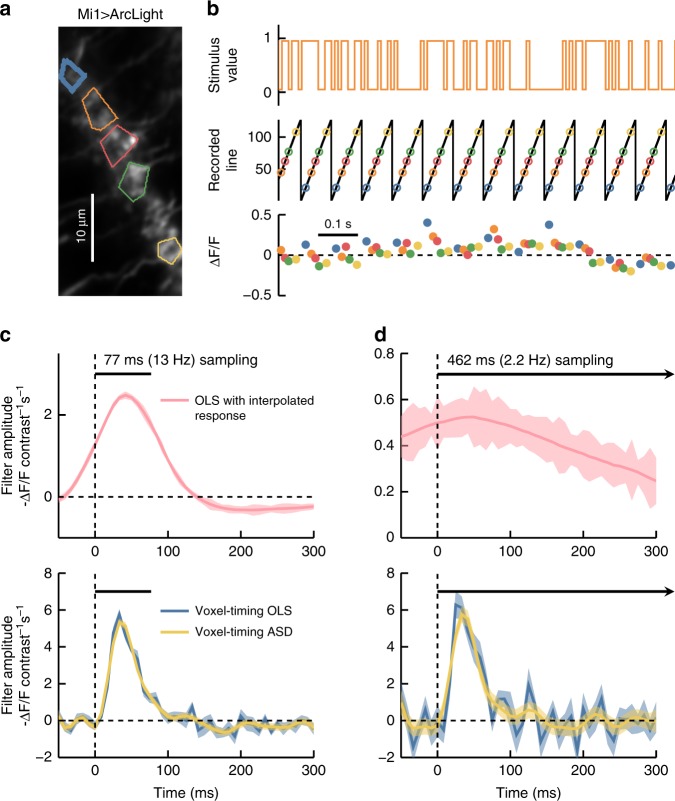
Voxel-timing voltage filters from *Drosophila* visual neurons. **a** Image of Mi1 dendritic arbors expressing ArcLight. Five regions of interest (ROIs) corresponding to different cells are outlined with different colors. **b** A stochastic binary light intensity stimulus was presented to the fly, and updated at 120 Hz (top). The *y*-position of the scan oscillates at the frame rate (~13 Hz) and ROI fluorescence is captured at intervals shown with colored circles (middle). Colored dots indicate the fluorescence and capture time for the five ROIs (bottom). **c** A best-fit filter (receptive field) for a sample ROI (thick blue line in **a**) was extracted from the 13 Hz data upsampled to 120 Hz through linear interpolation (top). This filter describes the weighting of inputs that best predicts the interpolated indicator response. This filter is limited by the timescale of the 13 Hz sampling interval (black bar). Best fit filters were also extracted from the same underlying data using the voxel-timing method in conjunction with OLS and ASD (bottom). Error bars throughout are ±1 SEM confidence intervals computed by bootstrapping response samples. **d** Best fit filters as in **c**, but using a subsampling of the original data in order to simulate a 2.2 Hz acquisition. When extracting the filter from an interpolated response (top), all high-frequency information is lost. Using the voxel-timing method (bottom), we recovered the high-resolution filter, though with more noise, since 6× fewer samples were used to compute this filter

To demonstrate this, we expressed ArcLight^51^, a fast reporter of membrane voltage (timescales ranging from ~10 to 80 ms), in *Drosophila* Mi1 neurons. These neurons respond quickly to light increments with a graded increase in membrane potential^40^. We presented flies with a ~10 min full-field binary visual stimulus^41^, updated stochastically at 120 Hz (Fig. 4a, b). We acquired two-photon images at ~13 Hz.

We first used the standard linear interpolation technique to upsample the responses to 120 Hz and find the OLS filter (Fig. 4c, top). The resulting filter was smoothed and its resolution limited by the 13 Hz acquisition rate. It was acausal, since the filter predicted that the cells would respond before a stimulus was presented.

We then used voxel-timing and OLS to extract a temporal super-resolution filter with the 120 Hz resolution of our stimulus (Fig. 4c, bottom). This filter exhibited dynamics faster than the imaging frame rate, and agreed with filters measured using fast linescan measurements (Supplementary Fig. 4) and with previous measurements of Mi1 voltage responses^13,40^. Using ASD to regularize the voxel-timing filter reduced noise slightly (Fig. 4c, bottom).

We further illustrated the independence of imaging frame rate and filter resolution by extracting filters from a simulated 2.2 Hz recording, by employing only two sampled measurements from each second of data. When this 2.2 Hz data was interpolated before fitting a filter, the filter was present, but substantially smeared in time (Fig. 4d, top). When instead we computed the voxel-timing filter, it retained the true high temporal resolution, but with increased noise due to fewer measured responses in the simulated dataset (Fig. 4d, bottom). Here, using ASD to regularize the filter reduced the noise substantially. When acquiring data to compute these receptive fields, the voxel-timing method eliminates the need for linescans or specialized, expensive hardware for fast acquisition rates.

### High-resolution glutamate filters from infrequent imaging

In principle, voltage could be measured in Mi1 neurons using electrophysiology^40^, rather than optical techniques. However, other quantities, like neurotransmitter concentrations, may only be measurable using optical indicators. An optical indicator for extracellular glutamate, iGluSnFR, is bright and responds on fast timescales of <20 ms^52^, making it a good candidate for high-resolution measurements of neurotransmitter dynamics at the surface of neurons.

**Fig. 5:**
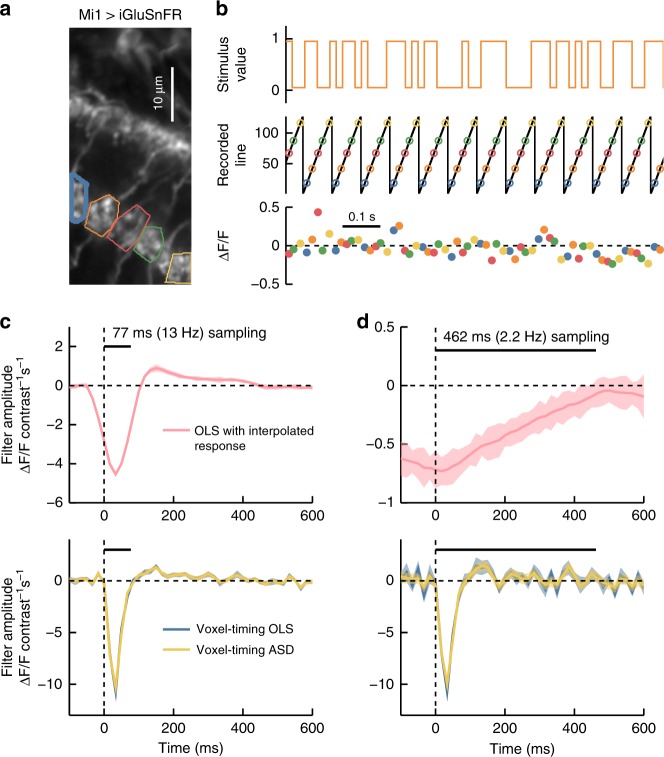
Voxel-timing glutamate filters from *Drosophila* visual neurons. **a** Image of Mi1 dendritic arbors expressing iGluSnFR. Five regions of interest (ROIs) corresponding to different cells are outlined with different colors. **b** A stochastic light intensity stimulus was presented to the fly, and updated at 60 Hz (top). The *y*-position of the scan oscillates at the frame rate (~13 Hz) and ROI fluorescence is captured at intervals shown with colored circles (middle). Colored dots indicate the fluorescence and capture time for the five ROIs (bottom). **c** A best-fit filter (receptive field) for a sample ROI (thick blue line in **a**) was extracted from the 13 Hz data upsampled to 60 Hz through linear interpolation (top). This filter is limited by the timescale of the 13 Hz sampling interval (black bar). Best fit filters were also extracted from the same underlying data using the voxel-timing method in conjunction with OLS and ASD (bottom). Error bars throughout are ±1 SEM confidence intervals computed by bootstrapping response samples. **d** Best fit filters as in **c**, but using a subsampling of the original data in order to simulate a 2.2 Hz acquisition. Filters were extracted from an interpolated response (top) or using the voxel-timing method (bottom)

To show that one may use slow imaging rates to measure fast glutamate dynamics, we expressed iGluSnFR in the neuron Mi1 in *Drosophila*, and presented the fly with a stochastic binary stimulus (Fig. 5a, b). A primary presynaptic partner of Mi1 is the neuron L1^53^, which responds to light decrements^12^ and is glutamatergic^54,55^. The ON-responsive Mi1 neurons likely invert these OFF glutamate signals by expressing the glutamate-gated chloride channel, gluCl^54^, while also receiving other inputs^56^.

The glutamate receptive field in Mi1 dendrites showed OFF responses, consistent with synaptic release from L1 (Fig. 5c). When these measurements were made at 13 Hz and interpolated to compute the 60 Hz receptive field (Fig. 5c, top), the glutamate filters appeared to be too slow to account for the fast voltage response seen in Fig. 4. However, the true temporal resolution of the response became visible when the filters were computed using voxel-timing analysis (Fig. 5c, bottom). Moreover, as with the voltage signals, we were able to extract glutamate receptive fields with far slower acquisitions, so that 2 or 0.5 Hz volumetric imaging could in principle be used with this indicator (Fig. 5d).

Glutamate signals have timescales of <20 ms, so a traditional imaging procedure that interpolated measurements to obtain receptive fields would have to image at 50 Hz or above to take advantage of its fast kinetics. Our results make clear that such fast imaging is unnecessary, and high-resolution receptive fields can be obtained with far slower frame rates. This permits such receptive fields to be found with standard microscopes and with volumetric imaging.

### High-resolution filters from volumetric acquisitions in V1

**Fig. 6:**
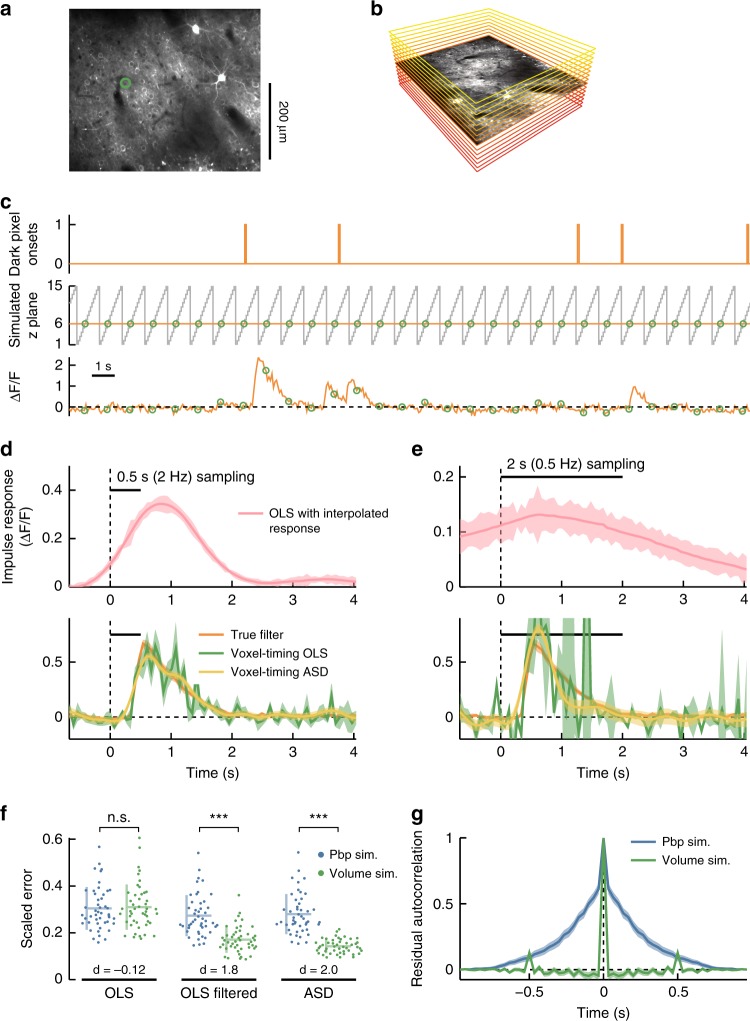
Precise temporal receptive fields from tree shrew V1 volumetric calcium imaging. **a** The original dataset recorded from neurons at 30 Hz at a single depth. A mean image of this recording shows the ROI used for filter extraction. **b** Simulated volumetric acquisition of 15 planes, one of which corresponds to the original recording. This plane is represented by the mean image from the original dataset, while other simulated planes are represented by colored rectangles. **c** Stochastic, sparse spatiotemporal noise was presented to the animal. Trace shows the onset times for dark stimuli at a single pixel (top). The set of planes in the volumetric dataset are represented in gray, while the static original plane is in orange (middle). We included response samples only where the simulated volumetric dataset measured from the true location of the neuron (green circles). Original calcium trace with circled simulated trace (bottom). **d** Impulse response of a single neuron in the simulated volumetric dataset using the interpolation method (top) and voxel-timing method (with and without ASD regularization) (bottom). Shaded patches are ±1 SEM calculated through bootstrapping. **e** Impulse responses calculated as in **d**, but from a simulated acquisition obtaining one volume every 2 s. **f** Distribution of errors in 47 cells from a simulated volume acquisition (2 Hz) and a simulated plane-by-plane (pbp) acquisition (30 Hz) with the same number of samples. Filters computed by OLS, by OLS followed by smoothing with a 7.5 Hz low pass filter, and by using ASD. Error is calculated as root mean squared deviation from the full dataset filter divided by the maximum value of that filter. Horizontal and vertical bars indicate sample mean and standard deviation, respectively. Effect size of using volumetric sampling versus plane-by-plane sampling is shown at bottom (Cohen’s *d*). Errors are reduced in volumetric vs. plane-by-plane in the smoothed and ASD cases (*p* < 1e–6), but not significantly different in the raw OLS case (*p* > 0.2), using a Wilcoxon signed-rank test. **g** Autocorrelation of residuals in the volumetric and plane-by-plane sampling cases

In cortex, cells in different layers play distinct processing roles^57,58^. Moreover, the spatial arrangement of receptive field properties in visual cortex has provided insight into the computational organization of cortical circuits^59–61^. In experiments that measure response properties of neurons at multiple depths, volumes are typically acquired plane-by-plane. That is, neurons in one plane are recorded at a high frame rate for some period of time, then the focal plane is moved, and then neurons from a different plane are recorded. In contrast, volumetric acquisitions sample an entire volume at a lower rate by measuring once from each plane before moving on to the next plane (Fig. 6a, b). The first method results in traces of neural activity that are sampled with high resolution (often at ~30 Hz), while the volume acquisitions result in traces of neural activity that are sampled infrequently. Below, we show that receptive fields may be obtained during volumetric imaging without loss of temporal resolution. Moreover, when the number of neural measurements is kept constant, receptive fields obtained through volumetric sampling can be of higher fidelity than those obtained from plane-by-plane sampling.

We analyzed two-photon GCaMP6s recordings of tree shrew V1 layer 2/3 neurons^59^. In the original dataset, frames were recorded at 30 Hz at a single plane. Sparse spatiotemporal noise stimuli were presented to the animal for ~20 min, with time courses qualitatively similar to sparse Poisson impulses (Fig. 1d). Using this data, we simulated a ~20 min 2 Hz volumetric acquisition with 15 planes per volume. In this simulation, each cell’s response was assumed to be measured in only one of 15 planes, while planes were acquired at 30 Hz (Fig. 6a–c). Since this simulated experiment would acquire data from 15 planes in each volume, it could characterize the response properties of up to ~15× more cells (with widely spaced planes) but would sample each cell 1× less frequently. We extracted receptive fields from OFF cells by correlating calcium activity to the onset of a dark pixel on the screen. When analyzed using linear interpolation between 2 Hz measurements, the OLS filter is smooth in time, lasting for ~2 s, and has acausal portions (Fig. 6d, top). In contrast, when the voxel-timing filter is computed, either with plain OLS or with ASD regularization, then the result is a filter that is very similar to the one obtained from 30 Hz sequential sampling (Fig. 6d, bottom). Thus, using the voxel-timing method, we recovered the same response dynamics from the infrequent volume acquisition as from the full, frequently sampled dataset.

We also simulated an even slower volume acquisition, in which the cell is measured once every 2 s. In this case, the OLS filter computed from a linearly interpolated response was very broad and smooth (Fig. 6e, top), but the voxel-timing ASD filter closely matched filter obtained from 30 Hz sequential sampling (Fig. 6e, bottom), despite being derived from 1/60th of the data, captured at 1/60th the frame rate. In this case, the cell was sampled too sparsely to generate a reliable OLS filter.

We finished by comparing volumetric sampling, in which each neuron was measured intermittently for the entire duration of the experiment, to plane-by-plane sampling, in which each neuron is measured frequently, but only for a short time. In this plane-by-plane simulation, the total number of samples from each neuron was the same as in the volumetric simulation. As expected, the noise magnitude in the volumetric acquisition filter was comparable to the noise in filters extracted in the simulated plane-by-plane experiment (Supplementary Fig. 5).

Interestingly, however, the method of infrequent volumetric sampling can be combined with other techniques to yield higher fidelity filters than traditional plane-by-plane sampling (Fig. 6f, g, see Supplementary Note 3). While the magnitude of errors in OLS filters derived from volumetric sampling is about equal to the magnitude of errors in plane-by-plane sampling, smoothing volumetric filters in time reduced these errors while smoothing plane-by-plane-derived filters had little effect (Fig. 6f). When ASD was applied to the both datasets, the volumetric filters consistently had higher fidelity. This is because errors in neighboring 30 Hz samples of the plane-by-plane simulation were more correlated with each other than those in neighboring 2 Hz samples in the volumetric simulation (Fig. 6g). Thus, residuals in filter estimates from the infrequent sampling were more independent. Temporal smoothing and ASD can take advantage of the independence of these errors to improve filter estimates. Thus, slower frame-rate, volumetric sampling of neurons does not limit the resolution of extracted temporal filters. Moreover, in some cases, it significantly improves filter estimates compared to equivalent frequent sampling.

## Discussion

Voxel-timing methods allow experimenters to use slow frame rate imaging data to extract filters whose temporal resolution does not depend on the rate of sampling of the neural response. This might surprise readers acquainted with the Shannon–Nyquist sampling theorem^18^. According to this theorem, when reconstructing a continuous signal from a series of samples, the sampling rate limits the frequencies contained in the reconstruction. Importantly, the theorem does not say that the higher frequencies do not exist in the sampled signal—they do. Rather, it says that those higher frequencies cannot be recovered through interpolation. The voxel-timing analysis applied here leverages this high-frequency information in infrequently-sampled neural responses to compute temporal super-resolution cross-correlations or filters. This method should be viewed as the correct way to compute cross-correlations with signals of this type, since it contains no implicit temporal smoothing of the true cross-correlation.

The approach presented here was proposed in fMRI studies^19^, and a similar approach was applied to remove heartbeat motion artifacts in fMRI data^20^. Voxel-timing analysis is also similar to spatial super-resolution methods in digital image processing. In those methods, several images of the same scene are acquired, offset from one another by less than a pixel-width. The different images are combined to generate a single image with resolution finer than that of the original images^23–25^. This approach has also been applied to image sequences, in which movies from multiple cameras with offset exposure times are combined to generate temporal super-resolution image sequences^62–64^. In all these cases, multiple sampled measurements are made of a single underlying signal, and it is the combination of measurements that increases the resolution. Here, we showed how applying this logic to neural imaging data permits filter resolution to be independent of imaging frame rate.

The method presented here is also similar to one previously used to precisely correlate calcium signals with behavioral outputs^65^. That study assumed instantaneous neural measurements and included data from many trials, which in principle allowed it to achieve high temporal resolution. Since it did not take into account the timing of measurements within each frame, systematic errors in the cross-correlation could arise. For example, in Fig. 4 of this manuscript, if a neuron was sampled at the end of each frame, but its responses were assigned to the beginning of each frame, the resulting filter would precede the true response by a full frame duration. Thus, if the latency between stimulus and response is important, it is critical not just to take into account short acquisition times, but also their exact timing within frames.

In this work, we were concerned with linear models like cross-correlation and filters, which represent the simplest models for relating neural responses to other variables. More generally, however, one may model responses as some function *ϕ* of the stimulus and some parameter set **θ**: $r_{t_i} = \phi ({\mathbf{s}}_{t_i},{\mathbf{\theta }})$. As with linear models, one may fit such nonlinear models by ignoring all unmeasured responses. Thus, voxel-timing can fit nonlinear or stochastic models with temporal super-resolution^66,67^, including computing response-weighted stimulus covariance, analogous to spike-triggered-covariance^32,33,68^ (Supplementary Fig. 2).

Voxel-timing analysis finds the relationship between the activity of an optical indicator and a high-resolution variable. However, optical indicators exhibit complex and often nonlinear relationships with underlying cellular quantities of interest, such as spike times, membrane potential, or calcium concentration^51,69^. Many methods relate calcium traces to spike times by modeling the nonlinear transformations present in different indicators^70–74^. Spike-time estimation methods are distinct from the method described here, but are complementary to it. For instance, work in songbird has combined many trials of calcium indicator measurements aligned to the bird’s song to generate a high-resolution calcium response, and then inferred spike times relative to the song with a resolution of a few milliseconds^65^. Other work used two-photon calcium imaging in cortical neurons to find precise spike timing relative to repeated current injections^75^. Thus, after finding a high temporal resolution indicator filter, spike inference methods can determine the spiking patterns that produced that average indicator response.

Voxel-timing analysis yields the greatest gain in resolution when activity indicators and neural responses are much faster than the interval between neural samples. Fast optical indicators are increasingly common: voltage indicators can have timescales of <50 ms^13,51^ down to 1 ms^76^; genetically encoded calcium indicators have timescales of <200 ms^69,77^; and synthetic calcium indicators can have timescales of <10 ms^69^. Glutamate reporters have timescales of <20 ms^52^. With voxel-timing analysis, these fast indicators could be used with volumetric two-photon, confocal, or light-sheet imaging, which often acquire volumes at rates of ~10 Hz or less. Standard galvanometric two-photon microscopy with slow frame rates may also be used to acquire high-resolution responses from fast indicators.

Voxel-timing analysis could be applied to many high-resolution experimental variables. We focused on visual stimuli, which are frequently presented at frame rates of 60 Hz and higher. However, many behaviors are recorded at video rates of 30 Hz or higher, and these are frequently correlated with neural activity^14,65^. Experiments may also make electrical measurements during imaging experiments, for instance recording intracellularly from a single neuron, recording field potentials, or recording electromyograms, all with resolutions of 1 kHz or higher. These could all be related with high resolution to infrequently sampled neural imaging data.

When should one apply a temporal super-resolution technique to compute filters or cross-correlation kernels? If one already has infrequently sampled measurements of neural activity, then there is no downside to computing temporal super-resolution cross-correlations. They will be noisier than those computed using interpolation, but one may smooth them in time to trade off this noise against temporal resolution (Fig. 3). One may also choose to recover the imaging-rate filter resolution by explicitly performing the smoothing that is implicit when responses are upsampled by interpolation. This manuscript provides code to easily apply voxel-timing methods to existing data.

In designing new experiments, there are many trade-offs to consider^78^. This method shows that the resolution of the receptive field or cross-correlation with a high-resolution variable is not limited by the response measurement intervals, and should not be considered a trade-off when low frame rates are used (Figs. 4–6). For instance, a one-hour experiment could obtain 10 min recordings from each of six planes at 12 Hz. Or it could measure volumetrically, acquiring all six planes sequentially at 2 Hz for the full hour. In both cases, the number of samples of each neuron is the same, and the extracted filters could have identical temporal resolution. However, under some conditions, filters may be better estimated when neurons are sampled infrequently rather than frequently in time (Fig. 6f, g). In designing volumetric experiments, the limits on the temporal resolution of the filter are the resolution of the stimulus and the duration of each neural measurement within the volume (see Supplementary Note 1). Thus, it is advantageous to match the integration time of each neural measurement with the timescale of the indicator or response kinetics, and one need not necessarily focus on maximizing sampling rates.

One drawback of slow imaging is that correlations between pairs of neural signals have a temporal resolution limited by the frame rate, since the relative lag between measurements of different neurons is fixed by their relative positions in the image or volume. This means neuronal cross-correlations are limited by the frame rate even though correlations with stimuli and behaviors are not. A second drawback of slow frame rate imaging is that individual responses to stimuli may be missed entirely, making it more difficult to examine trial by trial variation, which is often present in cortex^79^.

Voxel-timing analysis for filter extraction is compatible with many imaging modalities, neuron types, and optical indicators. It can be used to find correlations with many experimental variables measured on fast timescales: stimuli, electrical measurements, or behavioral outputs. This analysis employs straightforward mathematics, making it particularly easy to apply. Because it is an analysis method, it requires no new hardware and can be applied retrospectively to already-acquired data. This method can therefore be broadly applied to investigate fast correlates of neural activity.

## Methods

### Simulation details

Figures 1–3 show simulations of stimuli and responses. In all cases, the response was the stimulus convolved with a linear filter, plus added noise. Below, we provide the linear filters used and the level of noise for each figure. Code is provided for each figure, as noted under “Code availability” below.

In Fig. 1, the true linear filter is

$$f\left( t \right) = \frac{1}{Z}\exp \left( { - \frac{t}{{\tau _1}}} \right)\sin \left( {\frac{{2\pi t}}{{\tau _2}}} \right)$$

where *τ*_1_ = 100 ms and *τ*_2_ = 50 ms, and *Z* normalizes the filter so that its mean squared value equals 1. The stimulus is Gaussian white noise updated every millisecond, while the response is sampled for 1 ms every 100 ms. Uncorrelated Gaussian distributed noise was added to the response so that the signal-to-noise was ~60, as measured by the peak response divided by the standard deviation of the noise.

In Fig. 2, the true linear filter is

$$f\left( t \right) = \frac{{\exp \left( { - \frac{t}{{\tau _1}}} \right)}}{{\tau _1}}$$

where $\tau _1 = 10$ timesteps. The stimulus is uncorrelated Gaussian white noise updated every timestep, and the response is sampled every 10 timesteps. No noise was added to this response.

In Fig. 3, the true linear filter is

$$f\left( t \right) = \left( {1 - \exp \left( { - \frac{t}{{\tau _1}}} \right)} \right)\left( {\frac{1}{{\tau _2}}\exp \left( { - \frac{t}{{\tau _2}}} \right) - \frac{t}{{\tau _3^2}}\exp \left( { - \frac{t}{{\tau _3}}} \right)} \right)$$

where *τ*_1_ = 20 ms, *τ*_2_ = 100 ms, and *τ*_3_ = 200 ms. Noise was added to the response measurements so that the signal-to-noise ratio of the response was 1, as measured by the standard deviation of the filtered stimulus divided by the standard deviation of the noise. The response was sampled for 10 ms every 500 ms. The stimulus was updated at 100 Hz. Best fit linear filters were obtained by OLS fitting, except in the case of the ASD regularization technique.

In all cases, the filters were causal, so that *f*(*t* < 0) = 0.

### Drosophila voltage imaging and glutamate imaging

Flies were grown on cornmeal food at 29 °C. Mi1 neurons expressing ArcLight were imaged in vivo in response to visual stimuli^41,80^ presented on a panoramic screen around the fly^81^. The genotype of the experimental flies was +/w^–^; +/+; UAS-ArcLight/R19F01-Gal4^51,82^ for voltage measurements and +/w^–^; +/+; UAS-iGluSnFR/R19F01-Gal4 for glutamate measurements^52^. Visual stimuli were binary, full-field stimuli updated stochastically at 120 Hz, so that the screen flickered between light and dark gray, with contrasts of ±0.9. For glutamate imaging, the update rate was 60 Hz, and the contrasts were either ±0.2 or ±0.9. This stimulus was presented for 10 min to extract all shown kernels. Images were acquired with ScanImage^83^ on a 2-photon microscope (Scientifica, UK).

Line scans of neural activity (Supplementary Fig. 4a, b) were acquired at 416 lines per second. Mean fluorescent intensity of ROIs was first downsampled to 120 Hz, and mild bleed-through from the visual stimulus was subtracted. (Downsampling makes the kernel estimation easier, since all frequencies in the downsampled response have non-zero amplitudes in the stimulus. Without downsampling, one must regularize the equations to obtain the filter.) Mean pixel intensity in regions of interest were converted to Δ*F*/*F* by computing a baseline fluorescent trace, *F*(*t*), equal to a single exponential fitted to the entire trace. This baseline fluorescence was subtracted from the ROI fluorescence time trace in the numerator, and then used as the denominator. Filters were obtained using OLS to find the linear weights of the stimulus that best predicted the response at each 120 Hz sample (Supplementary Fig. 4c).

Full frames of neurons were acquired at 0.6 ms per line, and ~13 frames per second. With the magnification used, each neuron was sampled over ~10–15 ms during the frame. After ROIs were defined by hand around Mi1 dendrites, the response of the neuron in each scanned line was computed, finding Δ*F*/*F* as in the linescan case. These were aligned with the stimulus (see Suplementary Notes 4 and 5) and used to find filters for each line of each ROI; these filters were averaged together to find the filter for the entire region of interest. We simulated ~2.2 Hz volumetric acquisition by using every 6th measurement of the response to compute the filters.

Here and later, error bars were determined by bootstrapping, because OLS error estimates appeared to underestimate the true error in linear kernels. Bootstrapped 1 SEM errors were computed using Matlab’s built-in bias-corrected bootstrapping function, drawing random sets of neuron measurements.

### Tree shrew V1 calcium imaging

Time traces of individual cell fluorescence values and the stimulus were a kind gift from D. Fitzpatrick, from previously published experiments^59^. The stimulus presented in this experiment was a sparse noise stimulus with a 5 Hz update rate, and two-photon frames were recorded at 30 Hz for ~30 min. In order to extract high-resolution filters from this low-resolution stimulus, we created a 30 Hz stimulus trace corresponding to the onset of dark pixels (i.e. the stimulus trace was equal to 1 if a dark pixel turned on during the 30 Hz two-photon acquisition and 0 otherwise). We extracted filters by reverse correlation to each pixel individually.

To evaluate the error due to subsampling, we found the root-mean-squared deviation of each subsample-extracted filter from the fully sampled extracted filter. We then normalized these by the maximum value of the fully sampled extracted filter to obtain a scaled error. Reported scaled errors are medians across all subsample phases for each of the 47 cells. Because the fully sampled filter is treated as the ground-truth filter, we chose cells with significant fully sampled filters. Cell selection was performed by calculating the correlation between the neural signal and the stimulus for every pixel and every offset, and summing across offsets, yielding an index of correlation for each pixel for every cell. We analyzed cells whose maximum pixel index was above 1.

### Reporting summary

Further information on research design is available in the Nature Research Reporting Summary linked to this article.

## Supplementary information

Supplementary Information

Reporting Summary

## Data Availability

Data and Matlab code are available to generate all figure panels in Figs. 1, 2, 3 and 4. Code is also included to compute linear filters and cross-correlations from responses measured at arbitrary times relative to a stimulus with high temporal resolution. This code is available at: http://www.github.com/ClarkLabCode/FilterResolution
