# Supplementary material for: Using slow frame rate imaging to extract fast receptive fields
Source: Nat Commun. 2019 Oct 31;10:4979. doi: 10.1038/s41467-019-12974-0 (PMC6823504; doi:10.1038/s41467-019-12974-0)
Supplement: Supplementary file 1 — Supplementary Information [file 41467_2019_12974_MOESM1_ESM.pdf]

Supplementary Figures

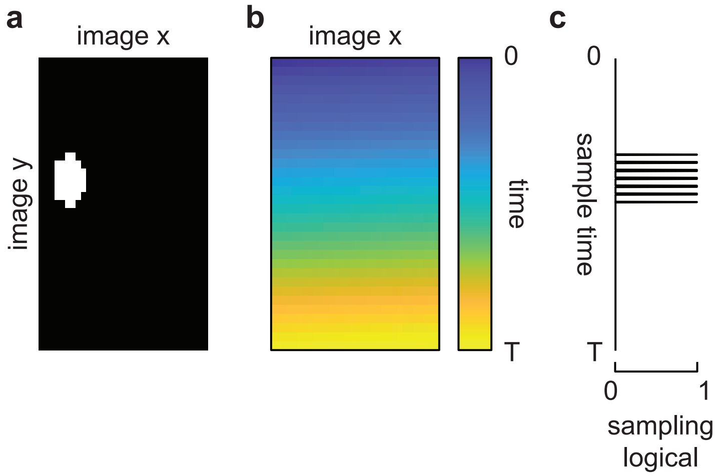

Supplementary Figure 1. Realistic ROI sampling in raster imaging.

(**a**) Individual neurons are typically contiguous regions of interest (ROIs) within an image, represented by the white region.

(**b**) In many imaging modalities, lines or planes are acquired sequentially during the acquisition time, from 0 to T. False color indicates relative time of rastered voxel acquisitions.

(**c**) Sampling logical for the ROI during the frame acquisition time. A sampling logical of 1 indicates that the neuron was being measured at that time. One could consider each pixel in the ROI as an independent measurement, or each line as a measurement. One may also consider the entire region of interest to be measured simultaneously within the longer image acquisition rate. Guidance on how to choose between these options is provided in **Supplementary Note 2**.

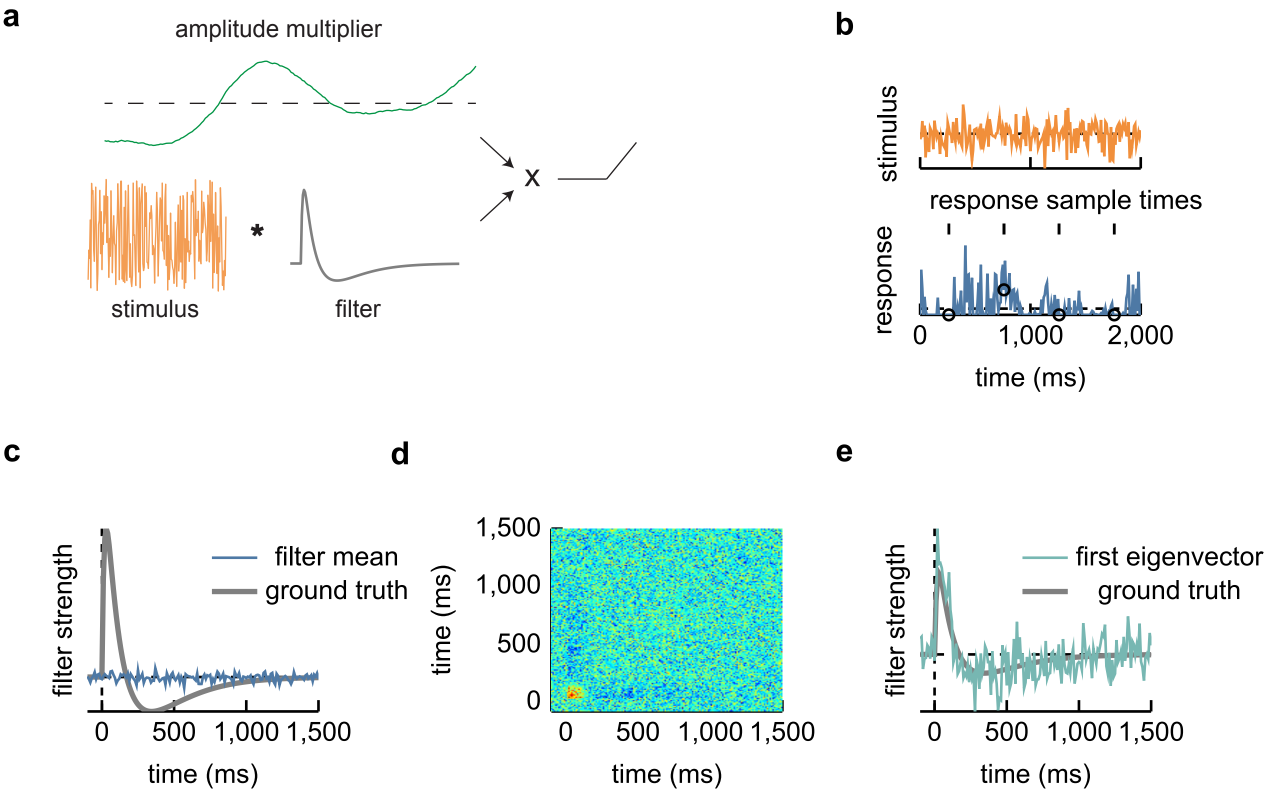

Supplementary Figure 2. Sparse sampling is compatible with second order methods.

(a) Schematic of the modeled cell. A slowly-varying random amplitude (top) is multiplied by the convolution of the stimulus with a filter. The result is passed through a rectifying nonlinearity.

(b) Stimulus and response for these numerical experiments. The stimulus was updated from a Gaussian distribution every 10 ms and a response was generated for every sample. Responses also included additive white noise, with a signal-to-noise ratio of 1. The response was sampled every 500 ms.

(c) The first order filter analysis presented in Figures 1 and 2 cannot capture the true filter because the amplitude randomly flips from positive to negative.

(d) The correlation structure of the stimulus preceding each response, weighted by the response. This is equivalent to spike-triggered-covariance for a continuous response[1,2], and reveals structured correlations in the stimulus that excite the simulated cell.

(e) The first eigenvector of the response-weighted correlation matrix corresponds to the true filter.

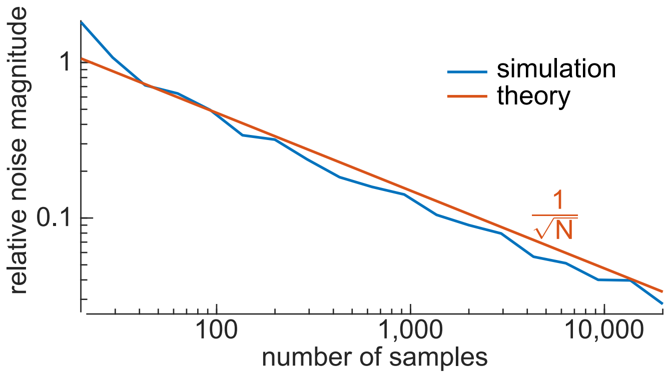

Supplementary Figure 3.

Increasing the number of measured response samples decreases the noise in the filter estimate. The decrease in noise follows a $N^{-1/2}$ trendline, where N is the number of samples.

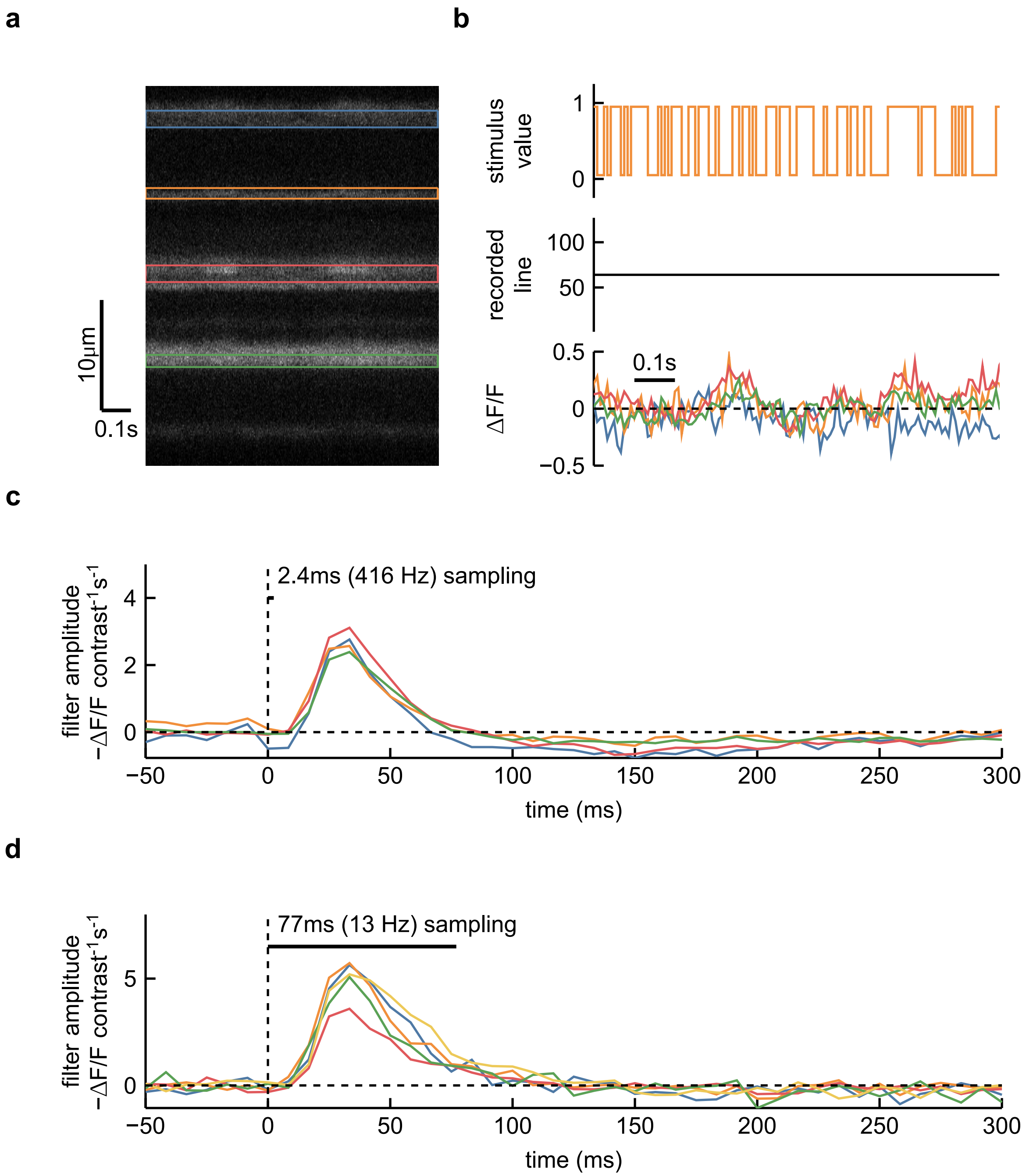

Supplementary Figure 4. Line scans of Mi1 neurons expressing ArcLight reveal fast timescale filters.

(**a**) Kymograph showing the intensity over time of line scans of Mi1 neurons expressing ArcLight. 1 second of data is shown; colored rectangles show regions of interest (ROIs).

(**b**) Stochastic, uncorrelated, binary full-field visual stimuli were presented to the fly, updated at 120 Hz (*top*). In line scan imaging, the y-position of the acquisition is constant throughout

the experiment (*middle*). Fluorescence traces of each ROI show responses to the flickering stimulus (*bottom*).

(**c**) Linear filters extracted from the line scans show that Mi1 responds to light increments within tens of milliseconds and provide a benchmark filter computation with high temporal resolution. Colors match the ROIs in (**a**) and (**b**).

(**d**) Linear filter extracted from the 13 Hz 2D acquisition in **Figure 3**. Colors match the ROIs in **Figure 3ab**.

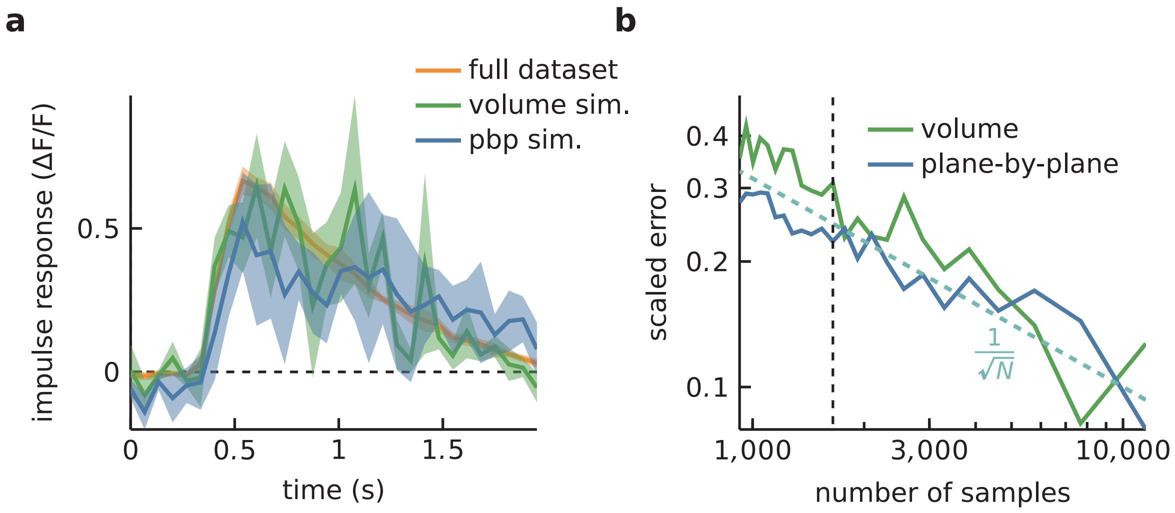

Supplementary Figure 5. Comparing volumetric to plane-by-plane sampling conditions in V1 calcium imaging.

(**a**) A baseline kernel for an example neuron may be extracted by using all of the data acquired at 30 Hz. It may be compared to the kernel computed from the simulated volume acquisition (2 Hz) and to the kernel computed from the simulated plane-by-plane (pbp) simulation acquired at 30 Hz. The number of samples in the plane-by-plane simulated acquisition matches the number in the simulated volume acquisition.

(**b**) For the example neuron, mean error for volume and plane-by-plane sampling protocols as a function of the number of samples. Error is calculated as in **Figure 6F**. The sloped dashed line shows a power law of $1/\sqrt{N}$ where N is the number of samples. The vertical dashed line indicates the simulated volume rate of 2 Hz.

Supplementary Note 1

In this note, we provide a short proof that the resolution of the cross-correlations between variables does not depend on how frequently one of the variables is sampled. This also derives expressions showing how the measured cross-correlation relates to the stimulus autocorrelation and the sample integration windows.

Neural responses to stimuli can be uniquely described by a series of Wiener kernels[3], which is similar to a Taylor expansion of a functional. Here, we examine the linear component of that series: the first order Wiener kernel. In continuous time, we wish to model the response, $r(t)$, as the convolution of the first order kernel, $f(t)$, with the stimulus, $s(t)$:

$$r(t) = \int_{-\infty}^{\infty} dt' f(t') s(t - t') = (f * s)(t) \tag{1}$$

where $f(t) = 0$ when $t < 0$, so that the filter is causal. The filter $f(t)$ includes the neural dynamics and the dynamics of the optical indicator used. One may compute a filter estimate $\hat{f}(t)$ by finding the reverse-correlation of the stimulus with the response. The expected value of some quantity, $v(t)$, is computed as a normalized integral over all time:

$$\langle v(t) \rangle_t = \lim_{T \to \infty} \frac{1}{T} \int_{-T/2}^{T/2} dt\, v(t) \tag{2}$$

Then, the estimate of the filter is

$$\begin{aligned} \hat{f}(t) &= \frac{1}{\sigma^2} \langle r(t') s(t' - t) \rangle_{t'} = \frac{1}{\sigma^2} \langle (f * s)(t') s(t' - t) \rangle_{t'} \\ &= \frac{1}{\sigma^2} (f * \langle s(t') s(t' - t'') \rangle_{t'})(t) \end{aligned} \tag{3}$$

or

$$\hat{f}(t) = \frac{1}{\sigma^2} (f * C_{ss})(t) \tag{4}$$

where σ^2 is the variance of $s(t)$, and C_{ss} is the (symmetric) autocorrelation function of the stimulus:

$$C_{ss}(t) = \langle s(t') s(t' - t) \rangle_{t'} = \langle s(t') s(t' + t) \rangle_{t'} \tag{5}$$

If $s(t)$ is uncorrelated and Gaussian distributed, then this method of computing $\hat{f}(t)$ yields an unbiased estimate of $f(t)$ even if a point nonlinearity acts on the filtered stimulus[4]. If the stimulus autocorrelation has structure in time, then $\hat{f}(t)$ is the true filter convolved with that autocorrelation function, $C_{ss}(t)$. If that autocorrelation function is known, then the filter may be deconvolved, an operation that is equivalent to OLS fitting of the filter.

In a real experiment, instead of the true $r(t)$, we instead measure $\bar{r}(t)$, which is the true response sampled periodically, with each sample integrating over the voxel integration time, defined by $h(t)$:

$$\bar{r}(t) = (r * h)(t)c(t) = (f * s * h)(t)c(t) \tag{6}$$

where $c(t) = \sum_{n=-\infty}^{\infty} T\delta(t - nT)$ is a Dirac comb function with period T, and $\langle c(t)\rangle_t = 1$. This expectation value of the comb function means that the measured response doesn't depend on the sampling interval. The sampling function, or voxel integration window, $h(t)$, is typically a boxcar function. One may now find the new cross-correlation estimate of the filter, $\bar{f}(t)$, using the sampled $\bar{r}(t)$:

$$\bar{f}(t) = \frac{1}{\sigma^2}\langle (f * s * h)(t')c(t')s(t' - t)\rangle_{t'} = \frac{1}{\sigma^2}(f * h * C_{ss})(t) \tag{7}$$

The estimate of the filter is the true filter convolved with the sampling function of the response, $h(t)$, and the stimulus autocorrelation, C_{ss}. This is because $\langle c(t')s(t')s(t' - t)\rangle_{t'} = C_{ss}(t)$ when the stimulus is time translation invariant, since the comb function just samples the stimulus at specific times. The measured filter is the true filter convolved with both the voxel integration window and the stimulus autocorrelation function, so the temporal resolution with which the filter is measured is determined by the longer timescale of the two. Note that the temporal resolution does not depend on the frame rate denoted in $c(t')$. If the response sampling function $h(t)$ and the stimulus autocorrelation function $C_{ss}(t)$ are both much faster than the true filter, $f(t)$, then $\bar{f}(t)$ is a good estimate of the filter, independently of how frequently the response was sampled. More samples will reduce the estimation error, but the resolution is independent of the sampling interval, T.

If the response measurement $\bar{r}(t)$ is linearly interpolated between samples to upsample it to the rate of the stimulus, this operation is equivalent to convolving $f(t)$ with an additional triangle filter, $R_T(t)$, which has a duration of the sampling interval, T. Then,

$$\bar{f}(t) = \frac{1}{\sigma^2}(f * h * C_{ss} * R_T)(t) \tag{8}$$

and now the filter estimate is convolved with a broad filter $R_T(t)$. Since $R_T(t)$ depends on the sampling frequency, $1/T$, it is much broader than the voxel integration window $h(t)$. Thus, interpolating reduces the temporal resolution of the filter estimate, potentially by a large degree for low sampling rates.

Supplementary Note 2

In practice, a stimulus is typically discrete and can often be made uncorrelated between samples. The discrete elements of the stimulus can be much faster than the sampling rate of the response. If the responses can be sampled over a short duration, then the filter may be estimated with little temporal error, independently of the sampling rate of the response. With discrete inputs, one can use OLS to solve for the filter estimate $\bar{f}$. That is, one has discrete stimulus s_t, with measurements of s at every time t. The response is measured at times t_i, so that one has an

associated set of responses, r_{t_i}. Then one wishes to construct a weighting function to best predict r_{t_i} from the n stimulus samples that preceded it, represented as a row vector $\boldsymbol{s}_{t_i}$ (see **Fig. 2**). This may be easily set up as a least-squares regression:

$$\begin{pmatrix} r_{t_1} \\ r_{t_2} \\ \cdots \\ r_{t_N} \end{pmatrix} = \begin{pmatrix} \mathbf{s}_{t_1} \\ \mathbf{s}_{t_2} \\ \cdots \\ \mathbf{s}_{t_N} \end{pmatrix} \boldsymbol{\beta} \tag{9}$$

where $\boldsymbol{\beta}$ is a vector with n elements and there are N measured responses. If $N > n$, then $\boldsymbol{\beta}$ may be fit using ordinary least-squares (OLS) regression so that it is the best fit weighting of the vectors $\boldsymbol{s}_{t_i}$ to predict the responses, r_{t_i}. The solution of this least squares regression is:

$$\widehat{\boldsymbol{\beta}} = (\mathbf{S}^{\mathrm{T}}\mathbf{S})^{-1}\mathbf{S}^{\mathrm{T}}\mathbf{r} \tag{10}$$

where the matrix $\mathbf{S}$ is the stimulus matrix above and the vector $\boldsymbol{r}$ is the vector of measured responses. Thus, the OLS solution is the cross-correlation between the stimulus and the response ($\mathbf{S}^{\mathrm{T}}\mathbf{r}$) divided by the autocorrelation of the vectors $\mathbf{s}_{t_i}$ ($= \mathbf{S}^{\mathrm{T}}\mathbf{S}$)[5]. Thus, this weighting $\widehat{\boldsymbol{\beta}}$ is a discrete version of $\bar{f}$ computed in the continuous case above.

When very few samples exist, the matrix $\mathbf{S}^{\mathrm{T}}\mathbf{S}$ may not be full-rank, in which case pseudo-inverses may be used. In cases where it is full-rank and well-conditioned, it is advantageous to use the actual covariance matrix of the applied stimuli rather than the long-time average covariance matrix[6]. If the attempted resolution is higher than the correlation time of the stimulus, then this matrix can be ill-conditioned, with zero or near-zero eigenvalues. In this case, one is attempting to deconvolve the stimulus autocorrelation, which may require strong regularization.

There remains the question of how to align the responses measured in the image with the discrete stimulus. Here, one could analyze the responses of a neuron pixel by pixel, using the timing of each pixel. For typical imaging parameters, this would yield response timing with microsecond-level temporal resolution. Given the temporal dynamics of optical indicators, this is higher resolution than is useful. Alternatively, one could analyze each line of pixels through a neuron by averaging over the pixels in the line and approximating them as all being sampled simultaneously. More coarsely, one could average over the entire ROI and approximate it as having been sampled simultaneously at the center of the ROI. These give similar results as long as the time window of averaging response pixels is not much longer than the stimulus frame rate or stimulus autocorrelation time. In Figures 4 and 5, we considered neural signals line by line; in Figure 6, they were considered plane-by-plane. If the response is measured during multiple time bins of the stimulus, it may be represented in more than one sequential element of the discretized response. This formulation is still compatible with the solution above.

In both the continuous and discrete cases, cross-correlations between the response and a stimulus were used to estimate receptive fields, but the logic holds for correlations between the response and any other variable that is not phase-locked to the sampling period, *T*. For instance, behavioral measurements like licking, whisking, and locomotor outputs, or fast neural measurements like EEG or LFP could be correlated in this way. Similar logic also applies to

fitting other nonlinear models, beyond the linearly weighted receptive field treated here. Such models would relate the measured responses r_{t_i} to the stimulus vectors $\boldsymbol{s}_{t_i}$.

Supplementary Note 3

The method presented here allows practitioners to obtain kernels with high resolution, but this is equivalent to fitting a model with a large number of parameters. Without a sufficient number of examples or regularization techniques, there can be significant noise in the estimated parameters. For a linear model, the signal-to-noise ratio (SNR) in the kernel elements can be estimated from the SNR of individual response measurements. To do this, we assume the noise is independent in each response measurement. Then the SNR, χ, of the temporal super-resolution cross-correlation is

$$\chi \propto \sqrt{N \frac{\Delta}{T}} \tag{11}$$

where N is the number of measured responses, Δ is the time-bin-width of the temporal super-resolution filter, and T is the interval between measurements. This formula is computed by recognizing that SNR increases with the square-root of the number of measurements in a bin. Compared to the frame-rate acquisition linear model (where $\Delta = T$), this formula permits one to compute how many more trials will be required to obtain cross-correlation elements with the same SNR.

Note that in practice, we do not find that errors are independent in each measurement of the response (**Figure 6g**). Rather, residuals correlate over time. When sampling infrequently, the residuals correlate less with each other than when sampling frequently. This allows smoothing in time or ASD regularization to improve kernel estimates more in the infrequently sampled case than in the frequently sampled case (**Figure 6f**).

One may estimate the error in a filter estimate by using bootstraps. Bootstrapped filters are computed for many different selections of neural responses (with replacement). From that distribution of synthetic filters, it is possible to estimate errors in the parameters of the true filter. We used this method to estimate errors in filters throughout the paper.

Supplementary Note 4

There are many use cases for voxel-timing analysis, but a common scenario will be aligning scanning microscopy with visual stimulus. In order to get scanline-level alignment between our imaging signals and visual stimuli, we used a channel of our imaging DAQ to acquire information about the stimulus during imaging sessions. If the stimulus information recorded in that channel is precise, then one may align the stimulus with each image with high precision. To obtain that high-precision information with visual stimuli, one may use a photodiode to record when certain frames of the stimulus were presented. This ensured high-precision alignment between our stimulus signal and fluorescence recordings.

When processing the data, we used the timing alignment channel to find which scanlines of the imaging raster corresponded to which frame of the stimulus. When performing this calculation, it is useful to know the acquisition time of each scanline. This can be computed as follows:

$$
\begin{aligned}
t_{scanline} &= T_{frame} * m + T_{scanline} * l \\
T_{frame} &= n_{scanlines} * T_{scanline} + T_{flyback}
\end{aligned} \tag{12}
$$

where $t_{scanline}$ is the acquisition time of the scanline, T_{frame} is the frame period (inverse of frame rate), m is the current frame number, $T_{scanline}$ is the line period, l is the current line number, $n_{scanlines}$ is the number of scanlines per frame, and $T_{flyback}$ is the flyback time for the scan to reposition itself at the end of each frame (not each line). In ScanImage 5, these are defined as follows:

T_{frame} = hSI.hRoiManager.scanFramePeriod
$T_{scanline}$ = hSI.hRoiManager.linePeriod
$n_{scanlines}$ = hSI.hRoiManager.linesPerFrame
$T_{flyback}$ = hSI.hScan2D.flybackTimePerFrame

Supplementary Note 5

Motion correction methods can affect this type of analysis because the pixel location in the frame determines the time at which it was sampled. Motion correction algorithms change the pixel location of neurons. In order to correctly define the timing of the sample of each neuron, the original, uncorrected pixel locations should be used for timing information. This would require a few simple additional steps in the analysis to locate each corrected pixel's original location. If the uncorrected locations are not used, the effect should be equivalent to a smoothing of the kernel in time on the timescale of typical spatial displacements of the motion.

Supplementary Citations

1 Mano, O. & Clark, D. A. Graphics processing unit-accelerated code for computing second-order wiener kernels and spike-triggered covariance. *PloS one* **12**, e0169842 (2017).

2 Sandler, R. A. & Marmarelis, V. Z. Understanding spike-triggered covariance using Wiener theory for receptive field identification. *J. Vis.* **15**, 16-16 (2015).

3 Wiener, N. Nonlinear problems in random theory. *Nonlinear Problems in Random Theory, by Norbert Wiener, pp. 142. ISBN 0-262-73012-X. Cambridge, Massachusetts, USA: The MIT Press, August 1966.(Paper)* **1** (1966).

4 Chichilnisky, E. A simple white noise analysis of neuronal light responses. *Network: Comput. Neural Syst.* **12**, 199-213 (2001).

5 Friedman, J., Hastie, T. & Tibshirani, R. *The elements of statistical learning*. Vol. 1 (Springer series in statistics New York, NY, USA:, 2001).

6 Korenberg, M., Billings, S., Liu, Y. & McIlroy, P. Orthogonal parameter estimation algorithm for non-linear stochastic systems. *International Journal of Control* **48**, 193-210 (1988).
